# How Is Rheology Involved in 3D Printing of Phase-Separated PVC-Acrylate Copolymers Obtained by Free Radical Polymerization

**DOI:** 10.3390/polym12092070

**Published:** 2020-09-12

**Authors:** Mario Iván Peñas, Miren Itxaso Calafel, Roberto Hernández Aguirresarobe, Manuel Tierno, José Ignacio Conde, Belén Pascual, Antxon Santamaría

**Affiliations:** 1 POLYMAT and Polymers and Advanced Materials: Physics, Chemistry and Technology Department, Faculty of Chemistry, UPV/EHU, Avda. Tolosa 72, 20018 San Sebastian, Spain; mpenas.3@alumni.unav.es (M.I.P.); roberto.hernandez@ehu.eus (R.H.A.); 2 ERCROS S.A., Innovation and Technology Department, Chlorine Derivatives Division, Diagonal 595, 08014 Barcelona, Spain; mtierno@ercros.es (M.T.); iconde@ercros.es (J.I.C.); bpascual@ercros.es (B.P.)

**Keywords:** rheology, PVC-Acrylate copolymers, phase separation, interlayer adhesion, 3D-printing

## Abstract

New auto-plasticised copolymers of poly(vinyl chloride)-r-(acrylate) and polyvinylchloride, obtained by radical polymerization, are investigated to analyse their capacity to be processed by 3D printing. The specific microstructure of the copolymers gives rise to a phase-separated morphology constituted by poly(vinyl chloride) (PVC) domains dispersed in a continuous phase of acrylate-vinyl chloride copolymer. The analysis of the rheological results allows the suitability of these copolymers to be assessed for use in a screw-driven 3D printer, but not by the fused filament fabrication method. This is due to the high melt elasticity of the copolymers, caused by interfacial tension between phases. A relationship between the relaxation modulus of the copolymers and the interlayer adhesion is established. Under adequate 3D-printing conditions, flexible and ductile samples with good dimensional stability and cohesion are obtained, as is proven by scanning electron microscopy (SEM) and tensile stress-strain tests.

## 1. Introduction

The growth of additive manufacturing (AM) techniques has produced a revolution in material processing methods since these technologies provide significant advantages over traditional processing techniques in terms of shape complexity, rapid prototyping, and so on [1,2,3]. In addition, additive manufacturing technologies offer the possibility of producing highly customized products for specific applications that can be designed locally and supplied throughout the world using the Internet [4]. Multiple additive manufacturing technologies have been developed for different materials, such as metals, ceramics, polymers and composites. However, owing to the specificity of each AM technology, available materials differ from traditional commodity materials [5]. Indeed, this is the case for polymers used for extrusion-based AM technologies, which are practically limited to semicrystalline rigid polymers, such as poly(lactic acid) (PLA), polypropylene (PP) or poly(ethylene glycol) (PETG), while the number of flexible alternatives is more restricted [2].

As a consequence, feedstock material development needs a thoughtful design capable of fulfilling the processing requirements of each AM technique. In the case of extrusion-based AM, the most popular technology is fused filament fabrication (FFF) where the printer is fed with a filament and forced through an extrusion nozzle. However, the need for filament fabrication is leading to an increase in the use of 3D printers fed with polymer pellets and driven by a single screw system. Although the end goal is similar in both technologies, i.e., to force the material through a printing nozzle, the flow characteristics are different. In the case of FFF, the flow resembles a plug flow where the filament acts as a piston in the solid state before melting. On the other hand, single screw systems produce a drag flow similar to conventional extrusion systems. The way the material is extruded, as well as the specific configuration of the printer, determine the printing requirements. In order to predict the printability, several works analyze the flow characteristics of different technologies, using rheological or finite element analysis approaches [6,7,8]. Even so, in both cases, the material interdiffusion becomes a crucial aspect to produce good material welding and, therefore, successful printing. Thus, in order to screen materials for extrusion-based AM, multiple models have been developed both to describe the interlayer welding at a molecular level [9,10,11,12]. However, such screening is still a challenge for complex fluids.

Among different materials of interest for extrusion-based AM, phase-separated polymeric materials attract both academic and industrial attention, due to their capability to combine very different characteristics, such improved elasticity, toughness, impact characteristics, and so on [13]. In the molten state, such polymers are complex fluids, eventually with self-assembled morphologies. This brings about a noticeable alteration of the dynamic viscoelastic behavior in the terminal or flow zone, with higher elastic modulus values than viscous modulus values [14]. Another rheological specificity is their viscoplastic behavior [15] that is to say an absence of the leveling off of the viscosity at sufficiently low shear rates typically observed in polymers. Due to its relevance and complexity, the performance of these materials in extrusion-based additive manufacturing should be analysed taking into account its rheological behaviour, both in extrusion and welding. The role played by interfacial tension between the phases is crucial, since it contributes to an increase of the elasticity of the melt [16]. This enhanced elasticity can make the material flow in the extrusion nozzle very difficult [17,18]. In the same way, the phase-separated morphology can also reduce the necessary chain mobility to produce the interlayer adhesion.

Thus, in this paper we analyze the printing implications of phase-separated polymer fluids, both during the material extrusion and the material welding, using different rheological techniques. In addition, we also analyse the mechanical performance of such materials and their dimensional stability. To do so, we present a series of new poly(vinyl chloride-acrylate) copolymers synthesized employing free radical polymerization. This brings about copolymers with phase-separated morphologies. As a matter of fact, it can be said that poly(vinyl chloride) (PVC) and its derivatives have not been used very much in additive manufacturing, so far, despite being one of the most used polymers in the world [19]. Research on this subject is, therefore, an issue of relevance.

## 2. Materials and Methods

### 2.1. Materials

The monomers used in the synthesis of copolymers, vinyl chloride (VCM), butyl acrylate (BA), 2-ethyl hexyl acrylate (EHA), and 2-heptyl propyl acrylate (2PHA) were supplied by Ercros (Barcelona, Spain), Arkema (BA and EHA, 99.5% purity) and BASF (2PHA). A fast initiator diisobutyryl peroxide (Trigonox^®^ 187-W40; DIBP), from Nouryon (Amsterdan, The Netherlands), was used as 40% aqueous emulsion.

The aqueous solution of suspending agents (5% *wt*/*wt*) was produced in situ by Ercros mixing the suspending agents and demineralized water. The suspending agent mixture consisted of two types of polyvinyl alcohol (PVA-1 and PVA-2), supplied by Nippon Gohsei, and hydroxypropyl methylcellulose (HPMC), purchased from DOW. PVA-1 was polyvinyl alcohol with a hydrolysis degree of 80% and a viscosity of 48 mPa·s (4% aq. sol, 20 °C) and PVA-2 was polyvinyl alcohol with a hydrolysis degree of 88% and a viscosity of 52 mPa·s (4% aq. sol, 20 °C). The HPMC used has a viscosity of 50 mPa·s. Finally, octadecyl 3-(3,5 -di-tert-butyl-4-hydroxyphenyl) propionate from BASF was used as an antioxidant.

### 2.2. Polymerization Procedure 

Poly (vinyl chloride-co-alkyl acrylate) copolymers were synthesized by free-radical polymerization in water suspension following the procedure described in the patent WO 2015/090657. The particularity of the synthetic method is based on the initiator dosage that permits accurate control of the reaction kinetics [20].

Polymerization reactions were carried out in a 300 L pilot plant stainless-steel reactor with two baffles type beavertail and an impeller type RCI (intense radial circulation). The reactor also was provided with a heating-cooling jacket, a reflux condenser, and a computerized control system. The reactor temperature was automatically controlled through a digital control system.

The polymerization procedure was carried out following the steps described below. First, under continuous stirring (90 rpm), the amount of demineralized water to reach a ratio water/monomer of 1.4 was loaded into the reactor. Afterward, the amount of the corresponding acrylate, detailed in Table 1, was loaded.

After the acrylate loading, a vacuum was applied to remove the oxygen present in the reaction medium and then 3000 ppm of the suspending agent mixture described in 2.1. was added. Immediately after that, the corresponding amount of VCM detailed in the Table 1 for each type of copolymer was incorporated into the reaction medium.

Then, the reaction mixture was heated, and when a temperature of 69 °C was reached, the stirring was increased to 180 rpm and the DIBP was continuously dosed at a constant flow rate.

The polymerization was maintained at this temperature until a drop in pressure of 7 bars was observed. This gives the VCM conversions, reaction times, and consumption of initiator included in Table 1.

At that moment, the dosing of DIBP was stopped and 100 ppm of antioxidant was added; the unreacted vinyl chloride was removed by depressurizing the reactor. The copolymer suspensions obtained were washed, centrifuged and the cake obtained was dried in a fluidized bed dryer.

### 2.3. Nuclear Magnetic Resonance (NMR)

Proton and carbon nuclear magnetic resonance spectra were obtained in a Bruker Analytische Messtechnik 500 spectrometer (Schramberg, Germany), at 500.13 MHz of resonance frequency, and using deuterated chloroform (CDCl_3_). A solution of 10 mg/mL and 50 mg/mL were prepared for ^1^H-NMR (nuclear magnetic resonance) and ^13^C-NMR, respectively. All of them were conveniently head treated and subjected to an ultrasonic agitation (30 min) to favour the dissolution of the copolymers. The composition of the copolymers was calculated from the integral areas of characteristics bands of acrylates and VCM comonomers.

### 2.4. Microscopy

Transmission electron microscopy (TEM) was used to analyse the microstructure of synthesized copolymers. Samples were trimmed at −60 °C using a cryo-ultramicrotome device, Leica EMFC6, Leica microsystems (Wetzlar, Germany), equipped with a diamond razor. The ultrathin sections (100 nm) were placed on 300 mesh copper grids. The surfaces were observed in a TECNAI G2 2o Twin (FEI, Hillsboro, OR, USA) operating at an accelerating voltage of 200 KeV in a bright-field image mode.

The welding zone of materials was analysed using scanning electron microscopy (SEM). TM3030Plus Tabletop Hitachi electron microscope (Hitachi High-Technologies Corporation, Fukuoka, Japan) was employed that operated at 15 kV in a standard (SD) observation mode. Before observation, the printed samples were cryo-fractured in liquid nitrogen and covered with a thin layer of gold using a Fine Coat Jeol Ion Sputter JFC-1100 (Peabody, MA, USA) to improve the electron conductivity during the SEM measurements.

### 2.5. Size Exclusion Chromatography (SEC)

The molecular weight distribution of synthesized copolymers was determined by size-exclusion chromatography (SEC) in a Waters 717 Autosampler chromatograph (Milford, MA, USA), consisting of a pump, a refractive index detector, and Waters Styragel (HR2, HR4, and HR6) columns. The analysis was carried out at 35 °C using tetrahydrofuran (THF) as eluent (flow rate of 1 mL/min). Measured distributions were referred to polystyrene narrow standards ranging from 580 to 395 × 10^3^ g/mole and corrected with the universal calibration using the Mark Houwink parameters for polystyrene: K = 1.58 10^4^ mL/g, a = 0.704.

### 2.6. Mechanical-Thermal Analysis

The glass transition temperatures of the synthesized copolymers were determined by dynamic mechanical thermal analysis (DMTA) measurements, conducted in bending mode in a Dynamic Mechanical Analyser, Triton 2000 DMA (Triton Technology, Mansfield, MA, USA). Rectangular samples were heated from 50 °C to 120 °C at a constant heating rate of 4 °C/min and a frequency of 1.0 Hz. 

### 2.7. Rheological Measurements

Extrusion flow experiments were performed in a Göttfert 2002 rheometer (Bunchen, Germany) using a capillary die with L/D = 30/1, at the temperatures and shear rates indicated in the Results and Discussion section. The viscosity curves, i.e., viscosity as a function of shear rate, η($\dot{\gamma }$), were obtained.

Small amplitude oscillatory shear (SAOS) experiments were conducted in linear viscoelastic conditions in an AR-G2 (TA Instruments, New Castle, DE, USA) rheometer, using 25 mm parallel plate geometry.

Stress relaxation experiments to obtain the relaxation modulus G(t) were carried out in an ARES rheometer (TA Instruments, New Castle, DE, USA) at the temperatures indicated in the text, using a parallel-plate fixture (12 mm diameter) and 1% of strain.

### 2.8. Printing Conditions

The printing specimens were obtained in a Voladora NX printer specially designed to work with pellets (Indart3D, Irun, Spain). The printing conditions were as indicated in Table 3 (Section 3.4.). A filament orientation of +45/−45° in a 3-lines contour, 100% infill and a 0.8 mm diameter nozzle were used in all cases.

### 2.9. Mechanical Properties of Printed Specimens

A universal testing machine Instron 4301 (Norwood, MA, USA) with a 5 kN load cell provided with an extensometer was used to perform tensile stress-strain tests. Type 5A tensile test specimens according to the UNE-EN ISO 527 standard obtained by compression moulding were employed. Previously the compression moulding specimens were prepared by adding to the products 6.6 phr of an organic stabilizer and 3 phr of epoxidized soybean oil, wherein “phr” is an abbreviation for “parts per hundred parts of resin”. The ingredients of the formulations were homogenized in a two-roll mill at 140 °C for 3 min. The obtained film was pressed at the same temperature in a hot plate press for 6 min at 60 kg/cm^2^ to obtain plates 2 mm thick, which were subsequently die-cut to the required shape.

## 3. Results and Discussion

### 3.1. Characterization

The macromolecular nature of synthesized copolymers was studied by SEC and ^1^H NMR spectroscopy. The results are shown in Table 2. SEC chromatography analysis revealed similar molecular weight distribution profiles for EHA- and 2PHA-based systems (Figure S1). However, PVC-BA showed a shoulder in the high molecular weight region. This effect has been explained considering that BA-based systems undergo both, intermolecular and intramolecular chain transfer reactions, during the polymerization [21,22]. In order to avoid differences regarding the molecular weight distribution, samples synthesized in the presence of different amounts of dodecanethiol (DDT) were included in the study. These new samples presented similar distributions and, accordingly, average molecular weights and polydispersities (Figure S1 and Table 2). The signal assignment for different copolymers (Figures S2 and S3) in the ^1^H NMR spectra revealed the incorporation of both co-monomer to the polymer structure and no lines corresponding to vinyl chloride (VC) and acrylate monomers were detected. In addition, the ^1^H NMR spectra allowed us to confirm that the respective acrylate concentrations of the synthesized copolymers were similar to those of the reaction mixture (Table 2).

**Figure 1:**
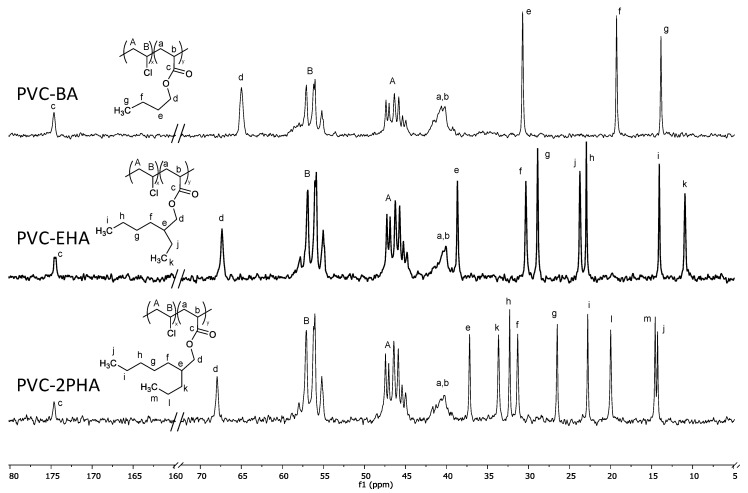
^13^C-NMR spectra of PVC-BA (top), PVC-EHA (middle) and PVC-2PHA (bottom). The lines assignment for all the copolymers is described at the continuation. PVC-BA copolymer (500 MHz, Cl_3_Cd, δ (ppm)): 174.7 (C=O (BA), 65.0 (OCH_2_ (BA)); 54.8–59.5 (CHCl (PVC)); 44.3–48.5 (CH_2_ (PVC)); 42.6–38.8 (CH, CH_2_ BA backbone); 30.7 (CH_2_ (BA sidechain)); 19.3 (CH_2_ (BA sidechain)) and 13.9 (CH_3_ (BA)). For the PVC-EHA and PVC-2PHA copolymers, the resonances of VCM units and the acrylate backbone are similar to PVC-BA ones. Even so, the PVC-EHA copolymer shows resonance peaks at: 174.4 (C=O (EHA), 67.3, OCH_2_ (EHA)), 38.7 (CH (EHA hexyl sidechain)), 30.3 (CH_2_ (EHA hexyl sidechain)), 28.9 (CH_2_ (EHA hexyl sidechain)), 22.9 (CH_2_ (EHA hexyl sidechain)), 14.1 (CH_3_ (EHA hexyl sidechain)), 23.1 (CH_2_ (EHA ethyl sidechain)), 10.9 (CH_3_ (EHA ethyl sidechain)). Finally, the PVC-2PHA copolymer shows peak resonances at (ppm): 174.6 (C=O (2PHA)), 67.9 (OCH_2_ (BA)); 42.6–38.8 (CH, CH_2_ (2PHA backbone)); 37.2 (CH (2PHA heptyl sidechain)); 31.3 (CH_2_ (2PHA heptyl sidechain)), 32.3 (CH_2_ (2PHA heptyl sidechain)), 22.8 (CH_2_ (2PHA heptyl sidechain)), 14.3 (CH_3_ (2PHA heptyl sidechain)), 33.6 (CH_2_ (2PHA propyl sidechain)), 20.0 (CH_2_ (2PHA propyl sidechain)) and 14.6 (CH_3_ (2PHA propyl sidechain)).

Further characterization was carried out by ^13^C-NMR to have a better understanding of the microstructure of synthesized copolymers. Figure 1 depicts a representative ^13^C-NMR spectrum of the poly(vinyl chloride)-butyl acrylate (PVC-BA), PVC-ethyl hexyl acrylate (PVC-EHA) and PVC-heptyl propyl acrylate (PVC-2PHA) copolymers.

Apart from confirming the synthesis of copolymers, ^13^C-NMR spectra can provide valuable information about the copolymer microstructure. Thus, in addition to the bands in the region between 54–60 ppm, that correspond to *mm*, *mr* and *rr* triads of VVV segments (V being an abbreviation of VC units) of PVC blocks, the copolymers show other ones of less intensity (see arrows). Those lines can be assigned to triads of VVA or AVA (where A stands for acrylate units), in view of our previous results [23] and literature results on vinyl chloride-vinylthiobenzene copolymers [24,25].

In addition, the broad band in the region between 38–42 ppm, which corresponds to the CH and CH_2_ carbons of the chain backbone of the acrylate, is characteristic of the copolymer formation. According to our previous work [16], the signals of CH and CH_2_ carbons of BA units in VB and BV monomer sequences shifted to higher values than the corresponding PBA homopolymer, BB sequences, which appear between 36.5–35.5 ppm. According to the spectra, alternative sequences of acrylate BV and VB sequences are predominant in the synthesized copolymers although the presence of BB sequences in PVC-BA cannot be discarded, mainly in PVC-BA samples (Figure S3).

**Figure 2:**
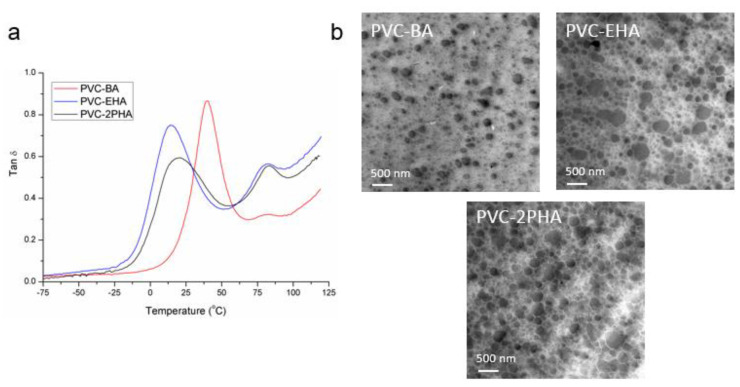
(**a**) Tan δ vs. temperature plots obtained at a constant frequency of 1 Hz and (**b**) transmission electron microscopy (TEM) images of synthesized copolymers.

The analysed co-monomer sequence has a strong influence on the physical and thermal properties of these copolymers, as revealed by temperature sweep experiments performed by DMTA. In Figure 2a the loss tangent (tan δ) is plotted against temperature and the observed maxima corresponds to the glass transition temperatures of the copolymers. Two glass transitions are seen for each copolymer, indicating a phase separation. The transition at 80 °C, which gives the lower tan δ peak, is close to the glass transition temperature of neat PVC (*T_g_* = 90 °C), suggesting that there are vinyl chloride-rich domains, in addition to a main random vinyl chloride-acrylate phase that gives the higher tan δ peak at temperatures 34 °C, 10 °C and 18 °C for PVC-BA, PVC-EHA, PVC-2PHA, respectively. These results are compatible with NMR spectra showing vinyl chloride sequences, in contrast to acrylate-vinyl chloride random distribution.

The phase-separated morphology suggested by NMR and DMTA results was confirmed by TEM analysis (Figure 2b). From the micrographs, vinyl chloride phases can be identified (black dots) dispersed along a continuous phase formed by acrylate-vinyl chloride copolymer. The average size of the nanodomains was 150 ± 40 nm in diameter for PVC-BA copolymer, 170 ± 70 nm for PVC-EHA copolymer and 220 ± 60 nm for PVC-2PHA copolymer. Those measurements were carried out on a minimum of 100 particles, prioritising the largest ones. It can be noted that the size of the vinyl chloride domains is smaller in PVC-BA copolymer than in the other copolymers, which is probably related to the lower second tan δ peak observed in DMTA results for this sample. This result can be related to the compatibility among PVC and acrylate homopolymers, where short side chain acrylates are expected to be more compatible with PVC than longer ones [26,27].

### 3.2. Rheological Study under 3D-Printing Conditions

**Figure 3:**
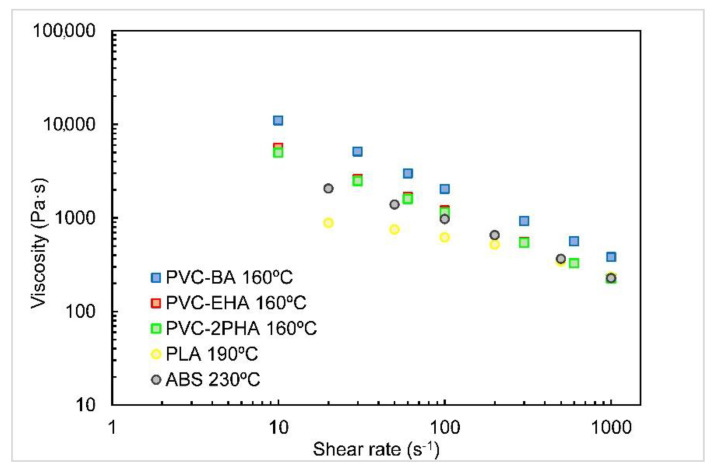
Viscosity vs. shear rate curves conducted at 160 °C for the copolymers indicated. The viscosity results of PLA and ABS at their corresponding processing temperatures are also included for comparison.

In order to correlate the rheological properties of the synthesized copolymers and the printing characteristics, samples were analysed by extrusion rheometry. As can be seen in Figure 3, samples show a highly pseudoplastic behaviour; the viscosity of PVC-EHA and PVC-2PHA copolymers is slightly lower than that of PVC-BA copolymer, due to the large aliphatic chains of the corresponding monomers. The viscosities of our copolymers were compared with those of very commonly used polymers in 3D printing, like poly(lactic acid) (PLA) and ABS, tested at respective typical printing temperatures. In view of these results, it was stated that the flowability of our copolymers was adequate for the extrusion in the nozzle.

**Figure 4:**
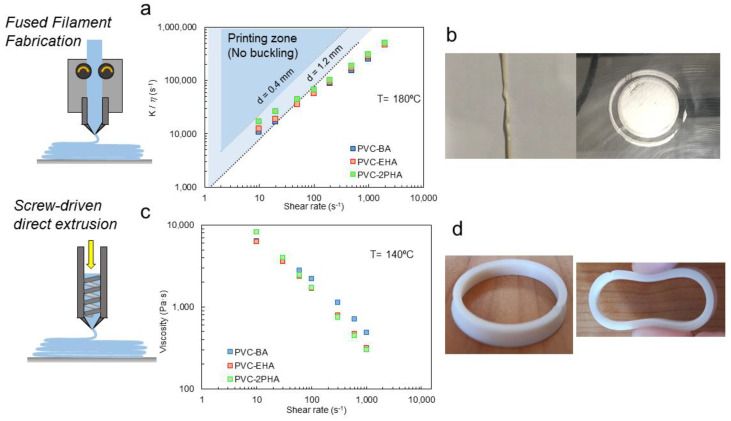
(**a**) Scheme of fused filament fabrication (FFF) technology; (**b**) *K/η* versus shear rate plots to ascertain buckling. The viscosity data are taken at 180 °C and the lines are drawn as explained in reference 23. Typical failure during FFF due to buckling; (**c**) Scheme of Screw-driven technology; (**d**) Viscosity plots at 140 °C and printed specimens using Screw-driven technology.

Therefore, we tried different 3D-printing technologies to confirm this statement. First, we tried conventional FFF technology. One of the most recurrent fails in this configuration is the filament buckling, especially in high viscosity materials [7,8,28]. This effect is governed both by the compression elastic modulus, *K*, of the material at room temperature and the viscosity, *η*, at the processing temperature. In fact, most of the materials fulfil the Venkataraman criterion, which relates the *K/η* ratio to the critical processing parameters for buckling [28,29]. In Figure 4, the Venkataraman plots at 180 °C for the synthesized materials are shown. None of the synthesized materials enters the printing zone for a 0.4 mm nozzle, but in the case of PVC-2PHA, the plot shows that it should be printable using a 1.2 mm nozzle and printing velocities corresponding to shear rates below 10 s^−1^. Moreover, an additional temperature increase should enhance the *K/η* ratio, but these plots could not be experimentally obtained due to degradation issues during rheological measurements. Notwithstanding the favourable conditions for FFF that can be found for the copolymers in this work, the fact is that none of the samples can successfully be printed using FFF, due to filament buckling which leads to the consequences seen in Figure 4b. In the case of PVC-2PHA materials, it was possible to print the first layer at 180 °C at very low printing velocities but the filament finally buckles. In addition, the increase of the temperature to 200 °C results in the material degradation during rheological measurement.

The issue of filament buckling in FFF of two-phase polymers has not been soundly considered, so far. Nevertheless, according to Venkataraman and the buckling criterion for an elastic column given by Euler’s analysis, buckling will occur when the pressure required for flow, Δ*P*, is higher than a critical buckling stress, *σ_cr_*. In the case of viscoelastic melts, Δ*P* is increased because additional force is necessary to overcome the elastic contribution. In heterogeneous systems, the flow of the melt is affected by the concentration, interaction, size, and morphology of the phases [30], leading eventually to buckling. Additional elasticity above close-packing of the grains can also be envisaged. In our samples, these phenomena are not very relevant, due to the uniform nanoscopic size of the respective dispersed domains. We consider that the actual problems for FFF printing arise from the high melt elasticity of the samples which, in turn, results from the interfacial tension between phases. This is considered in Section 3.3.

In order to overcome this effect, we decided to move from FFF technology to a screw-driven direct extrusion 3D printer. The absence of a filament eliminates the possibility of material buckling and the material viscosity mainly governs the material extrusion. Even so, the low differences among materials shown in the viscosity plots (Figure 3 and Figure 4c) resulted in no significant differences in their printability. In fact, by using this kind of printer, it was possible to process PVC-acrylate copolymers even at lower temperatures, as is observed in Figure 4d and Section 2.4. In our previous work [31], based on the printability of PVC/acrylate random copolymers, it was demonstrated that the copolymer with 40% acrylate content presented little mass loss after 45 min at 160 °C; this is much longer than the residence time (5–10 min). Furthermore, preliminary thermogravimetric (TGA) studies carried out heating at 10 °C/min show that these copolymers start to degrade above 180 °C, so we can assume that they do not suffer thermal degradation during screw-driven printing.

### 3.3. Influence of Viscoelastic Characteristics on 3D-Printing Performance

**Figure 5:**
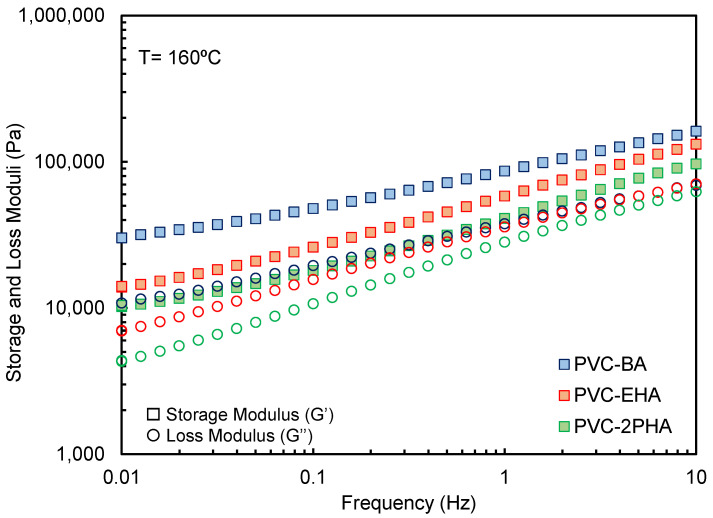
Elastic and loss moduli as a function of frequency for the indicated samples at a temperature of T = 160 °C.

In comparison to traditional feedstock materials for extrusion-based additive manufacturing, PVC-acrylate copolymers showed a flexible and highly elastic character in the molten state. As shown in Figure 5, all samples showed a predominantly elastic character as the elastic moduli (G′) is over the loss moduli (G″) for all the frequency region. This is more predominant for PVC-BA samples, where the G′ values at low frequencies are higher than those of PVC-EHA and PVC-2PHA in the same frequency range. Considering literature results on dynamic viscoelasticity of two-phase systems [32,33,34], we infer that the observed high elasticity values owing to the contribution of the interfacial elasticity, which is a consequence of the phase separation state of our copolymers, as is observed in Figure 2. In turn, the corresponding interfacial tension widens considerably the relaxation time spectrum, as compared to one phase systems such as homopolymers and random copolymers. The Cole-Cole plots, that is to say, the double logarithmic plots of the real part of the complex viscosity, *η*″, versus the imaginary part, *η*′, constitute a very suitable rheological method for the study of the widening of the relaxation time spectrum. In fact, in such plots an arc is systematically observed for one single-phase system, allowing a characteristic relaxation time to be defined as the inverse of the frequency at the maximum of the arc. However, in phase-separated or multiphase systems the arc disappears and, instead, two arcs or a straight line are detected.

**Figure 6:**
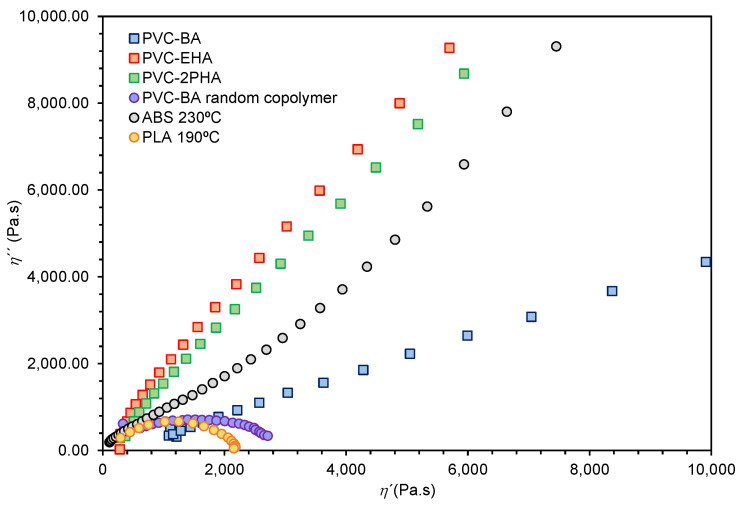
Cole–Cole plots of the phase separated PVC-acrylic copolymers showing straight lines as the frequency is decreased (right side). The results of one phase polymers, fully random PVC-BA copolymer and PLA, respectively, are shown for comparison.

In Figure 6, the Cole–Cole plots of our copolymers are presented and compared to a pair of single phase polymers, a fully random PVC-BA copolymer and poly(lactic acid) (PLA), both adequate for FFF [35,36]. Following general literature results, which stand for the different response of one phase and multiphase polymers, a single relaxation time in the terminal zone (inverse of the frequency of the maximum of the arc) is observed for both fully random PVC-BA copolymer and PLA homopolymer. In contrast, the influence of the interfacial relaxation, owed to phase separation, impedes the existence of a single relaxation time, leading to straight lines in Cole–Cole plots at low frequencies, as is observed for our phase-separated PVC-acrylic copolymers.

The effect of the interface, contributing to a high elasticity in the molten state, can alter the material performance in extrusion-based additive manufacturing, in particular in the case of FFF. It is known that the first normal stress N1 values, developed for instance in capillary flow, are directly related to the elastic modulus G′ [37]. Thus, according to the results of Figure 5, the N1 values of our samples should be very high, although determining the exact amounts is a difficult task, beyond the scope of this work. These results are related to the buckling observed in Section 2.2 taking into account that the entrance pressure drop, typically observed in the transition from the barrel to the die, is directly proportional to the first normal stress difference [38]. With these results in hand, we can assert that the buckling analysis based on *K/η* ratio (Figure 4) is not correct, since it is based on viscosity results obtained when ignoring the entrance pressure effect. Actually, when our very elastic copolymer melts are forced to pass from the barrel to the nozzle, pressures considerably higher than expected are required, because to make the polymer to flow it is necessary to overcome the pressure drop at the entrance. This is the same effect as in increasing the viscosity, obtaining lower *K/η* ratios than expected, which makes buckling to occur.

This reasoning is compatible with the analysis performed by some authors that introduce the material elasticity as a key parameter for describing the viscoelastic flow of polymer melts through the nozzle. As explained by Galindo-Rosales et al. [18], the generation of vortexes upstream in the flow reduces the effective nozzle diameter. This reduction of the effective diameter of the nozzle can lead to an increase in the printing pressure and, finally, to filament buckling. Certainly, this effect is not relevant in the case of screw-driven direct extrusion, as the screw can produce sufficient stress to overcome these effects and produce the material flow.

Therefore, we have to point out that due to the high melt elasticity of our copolymers it is necessary to use the screw-driven extrusion 3D.

**Figure 7:**
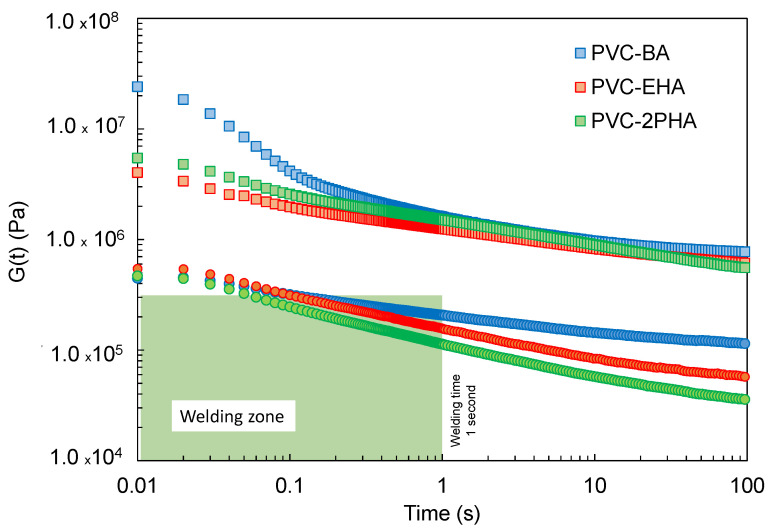
Stress relaxation experiments of synthesized copolymers at 40 °C (empty symbols) and 100 °C (filled symbols). The green area highlights the zone where the welding is expected. This zone is delimited by the Dahlquist criterion (3 × 10^5^ Pa) and a welding time of 1 s [10,40] (see text).

Regarding the interlayer adhesion, the viscoelastic characteristics of polymers directly affect the molecular interdiffusion necessary for successful welding. Although this adhesion is favoured for materials with a predominantly viscous behaviour at the welding temperature (G″ > G′), in our previous works we establish a simple methodology to qualitatively evaluate the adhesion performance based on the Dahlquist criterion, regardless their relative values of G′ and G″ [29,39]. Thus, stress relaxation experiments were performed at different temperatures (Figure 7).

According to the results shown in Figure 7, synthesised copolymers can be accomplished with the Dahlquist criterion at times below 1 s at 100 °C and, therefore, it is expected to have a good interlayer welding above this temperature. In contrast, the relaxation moduli of samples at 40 °C present higher values, which can impede the interlayer adhesion. Therefore, in order to produce sufficient interlayer welding, the temperature of the printing substrate was set around 100 °C.

### 3.4. Printing Study and Mechanical Properties of Tensile Test Specimens

Taking into account the rheological characterization explained in Section 3.2, the printing conditions of the synthesized copolymers were in each case optimized. In addition to extrusion flow requirements, the temperature of the heated printing bed was also optimized, due to its crucial relevance for interlayer welding. Both optimized parameters, printing, and bed temperature are summarized in Table 3 together with the maximum printing speed.

**Figure 8:**
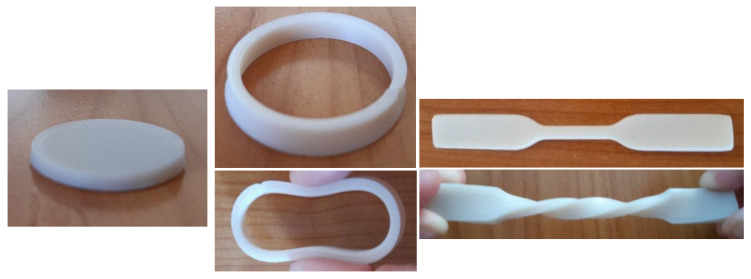
Printed 3D shapes using different PVC-Acrylate copolymers. (**a**) PVC-2PHA disk, (**b**) PVC-EHA ring; (**c**) PVC-BA tensile bar. The ductility of the samples is noticed by the procured crushing and twisting.

As shown in the table, the samples were printed at temperatures lower than 160 °C. In contrast, in a previous work PVC formulations with 30–40% of diisononyl phthalate (DINP) plasticizer were printed at temperatures above 190 °C using FFF technique [29]. Indeed, the difference in printing method, FFF for PVC/DINP formulations and screw-driven for PVC-acrylate copolymers, lies on the monophase character of the former, due to the phase separated nature of the copolymers. Under the printing conditions described in Table 3, it was possible to print different three-dimensional flexible shapes using synthesized copolymers as feedstock materials, as shown in Figure 8.

**Figure 9:**
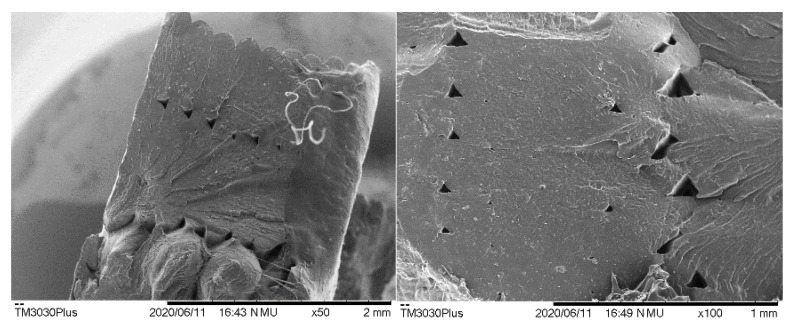
Scanning electron microscopy (SEM) microphotograph of consecutive layers of PVC-BA copolymer. The image on the left shows the arrangement of the contour lines. The image on the right corresponds to the inner section.

The printed specimens are very flexible and showed good dimensional accuracy, with a negligible shrinkage, ductility, and cohesive aspect. Concerning this last feature, interlayer welding was analysed by SEM (Figure 9) revealing a good adhesion between consecutive layers and thus confirming the soundness of the aforementioned viscoelastic criterion.

An analysis of the mechanical properties of printed samples is carried out to confirm the good interlayer adhesion observed by SEM, as well as to put in value the eventual applications of our copolymers.

**Figure 10:**
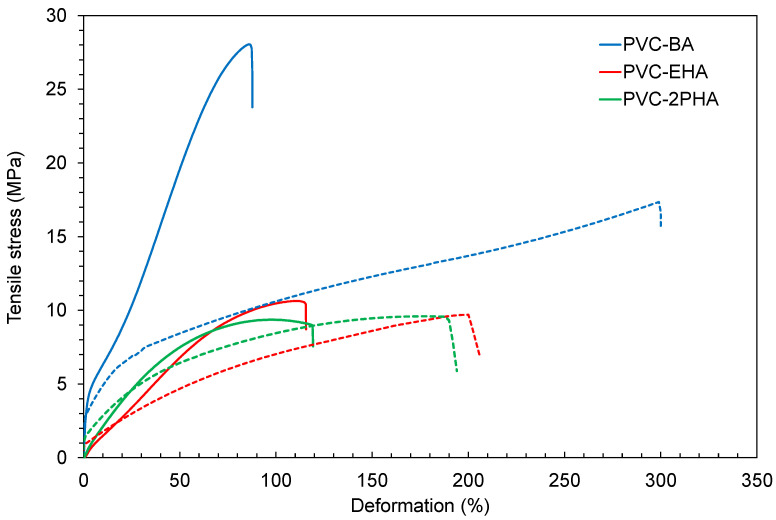
Tensile stress-strain results obtained as explained in the experimental part. Solid lines correspond to 3D-printing bars, whereas dotted lines correspond to bars procured by compression moulding.

The results of the 3D-printed tensile bars were compared with those of the tensile bars prepared by compression moulding, following the UNE-EN ISO 527 standard method typically used to test the final mechanical properties of commercial PVC and PVC derivatives. In Figure 10 the tensile stress-strain results of tensile bars are shown for the different copolymers; a summary of the results is shown in Table 4.

As can be seen, the copolymer that contains butyl-acrylate, PVC-BA, shows higher values of tensile strength in both specimens obtained by 3D printing and by compression moulding. This could be expected, considering the more rigid segments of BA (higher *T_g_* noticed by DMTA results, see Figure 2a when compared with EHA and 2PHA.

It must be mentioned that the elongation at break of printed specimens showed a remarkable decrease in the elongation at break (see Figure 10 and Table 4). The main reason for this reduction relies on the sensitivity of the elongation at break to the presence of voids produced by the layer-by-layer process, as depicted in Figure 9 [41,42,43].

Although our 3D specimens show suitable deformation at break and tensile stress values, a less-ductile behaviour than the samples prepared by compression moulding is noted due to the lower deformation at break. This is not unexpected, since the orientation produced during extrusion in the nozzle for 3D printing gives rise to an increase of the rigidity, i.e., lower deformation at break and higher tensile stress values.

The mechanical strength of specimens produced by 3D printing is always lower than that of the original material or that of moulded pieces [44,45,46]. Several efforts have been made in order to improve the mechanical properties of 3D-printing pieces by means of the printing parameters modification. However, the PVC-acrylate specimens obtained by 3D printing have shown at least equal, if not improved, properties which could represent a significant advance in this area.

## 4. Conclusions

PVC-acrylate flexible copolymers have been obtained by free radical polymerization, bringing about a pseudo-block microstructure, where poly(vinyl chloride)-r-(acrylate), as well as poly(vinyl chloride) microstructures, can be detected. This specific microstructure gives rise to a phase-separated morphology constituted by an acrylate-vinyl chloride-based continuous phase and PVC domains.

The rheological analysis of the copolymers shows that the viscosity values are similar to those obtained for polymers currently used in 3D printing, such as PLA and ABS, in the range of shear rates (10 s^−1^ to 1000 s^−1^) involved in the printing process. However, it is observed that buckling makes FFF impossible with these copolymers. Our hypothesis is that buckling is mostly due to the high pressure drop developed at the entrance of the nozzle, which leads to an increase in the apparent viscosity. Linking polymer structure with rheology and AM, we can assert that interfacial tension between phases causes an enhancement of melt elasticity (higher G′ values) which in turn leads to high pressures in the nozzle and, consequently, buckling.

On the other hand, considering the peculiar viscoelastic behaviour of our copolymers an approach to the liaison between the relaxation modulus and the interlayer adhesion is presented, which allows a viscoelastic map to be established for suitable welding.

Taking these copolymers as feedstock for use in a simple screw-driven 3D printer gives the opportunity to obtain a range of flexible objects with high toughness, proper dimensional accuracy, dimensional stability (absence of warping), and a cohesive aspect.

SEM analysis of the welding parts shows good adhesion between consecutive layers, which is compatible with the observed ductility of 3D-printed tensile bar specimens.

## Figures and Tables

**Table 1:** Reaction parameters.

Parameter	Units	BA	EHA	2PHA
Amount of acrylate	kg	22.7	23.1	22.0
Amount of VCM	kg	55.0	59.5	61.0
Amount of initiator	kg	0.680	0.714	0.722
Reaction time	min	243	148	300
VCM conversion	%	79	83	84

**Table 2:** Acrylate concentration in the reaction mixture and the synthesized copolymers. Average molecular weights and polydispersity index for each sample.

Sample	Acrylate Composition in the Reaction Mixture (%)	Acrylate Composition in the Copolymer (%) *	Mw ** (g mol^−1^)	Mn ** (g mol^−1^)	IP
PVC-BA	30	33	296,250	65,400	4.5
PVC-EHA	30	30	208,200	61,100	3.4
PVC-2PHA	30	34	188,800	56,550	3.3

* Calculated by ^1^H-NMR (nuclear magnetic resonance); ** Referred to polystyrene standards.

**Table 3:** Printing conditions in screw-driven direct extrusion for synthesized PVC-acrylate formulations.

Sample	Printing Temperature (°C)	Maximum Speed (mm/s)	Bed Temperature (°C)
PVC-BA	120–160	8	30–60
PVC-EHA	120–160	8	30–80
PVC-2PHA	120–160	8	30–60

**Table 4:** Summary of the tensile-stress results obtained from the data of Figure 10.

Properties	PVC-BA		PVC-EHA		PVC-2PHA	
	Compression Moulding	3D	Compression Moulding	3D	Compression Moulding	3D
Tensile stress (MPa)	17.3 ± 2.2	27.9 ± 0.3	9.7 ± 0.3	10.5 ± 0.1	9.6 ± 1.8	8.9 ± 0.7
Deformation at break (%)	292 ± 4	91 ± 3	204 ± 9	116 ± 6	188 ± 3	122 ± 6

## Supplementary Materials

The following are available online at https://www.mdpi.com/2073-4360/12/9/2070/s1: Figure S1: Size-exclusion chromatography (SEC) chromatograms for the synthesized copolymers, Figure S2: 1H-NMR spectra for synthesized PVC-acrylate copolymers (PVC-BA), as well as the corresponding line assignment, Figure S3: Comparison between ^13^C-NMR spectra for synthesized PVC-BA copolymers and PBA homopolymer.
