# Supplementary material for: How Is Rheology Involved in 3D Printing of Phase-Separated PVC-Acrylate Copolymers Obtained by Free Radical Polymerization
Source: Polymers (Basel). 2020 Sep 12;12(9):2070. doi: 10.3390/polym12092070 (PMC7569851; doi:10.3390/polym12092070)
Supplement: Supplementary file 1 [file polymers-12-02070-s001.pdf]

SUPPORTING INFORMATION

How is Rheology Involved in 3D Printing of Phase-Separated PVC-Acrylate Copolymers Obtained by Free Radical Polymerization

Mario Iván Peñas [1], Miren Itxaso Calafel [1,*], Roberto Hernández Aguirresarobe [1], Manuel Tierno [2], José Ignacio Conde [2], Belén Pascual [2] and Antxon Santamaría [1,*]

[1] POLYMAT and Polymers and Advanced Materials: Physics, Chemistry and Technology Department, Faculty of Chemistry, UPV/EHU, Avda. Tolosa 72, 20018 San Sebastian, Spain; mpenas.3@alumni.unav.es (M.I.P.); roberto.hernandez@ehu.eus (R.H.A.)

[2] ERCROS S.A., Innovation and Technology Department, Chlorine Derivatives Division, Diagonal 595, 08014 Barcelona, Spain; mtierno@ercros.es (M.T.); iconde@ercros.es (J.I.C.); bpascual@ercros.es (B.P.)

* Correspondence: itxaso.calafel@ehu.eus (M.I.C.); antxon.santamaria@ehu.eus (A.S.)

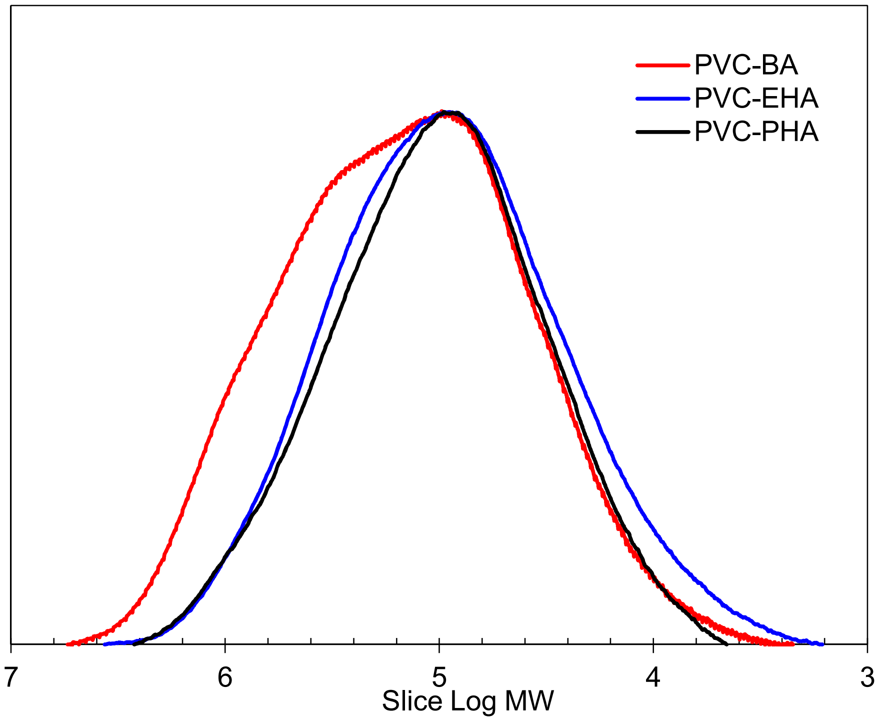

Figure S1. Normalized SEC chromatograms for the synthesized copolymers.

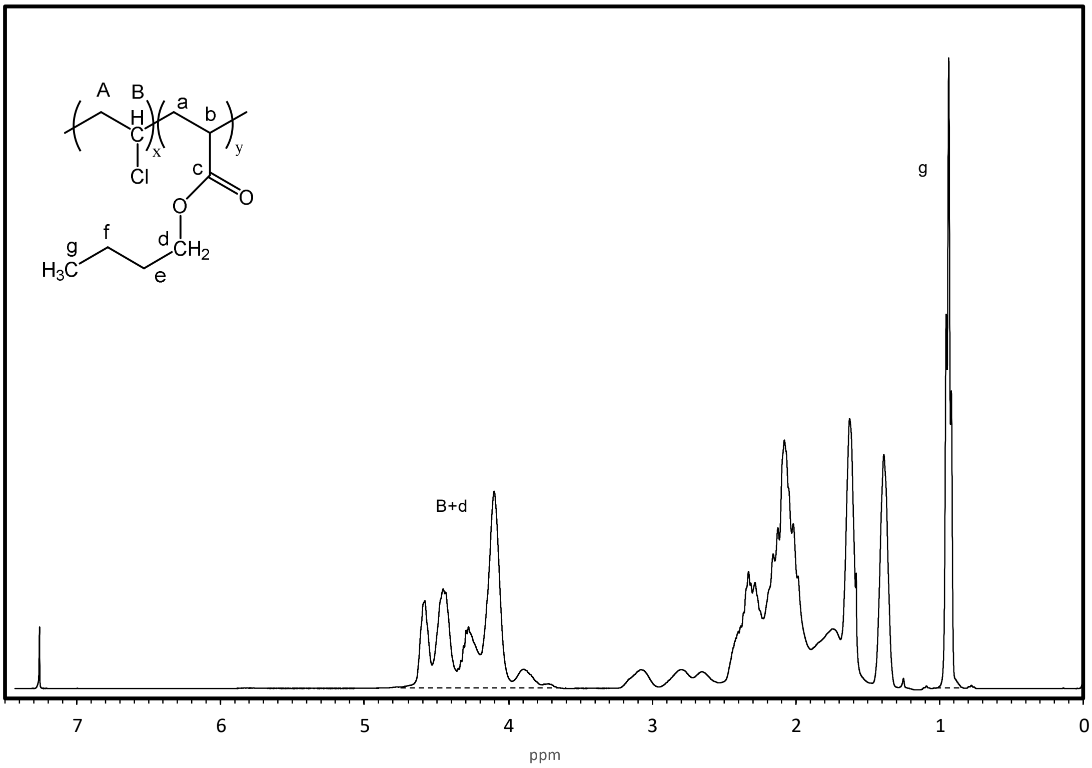

Figure S2. ^{1}H-NMR spectra for synthesized PVC-acrylate copolymers (PVC-BA), as well as the corresponding line assignment. The copolymer composition was calculated from the integral areas of characteristics bands of BA and VC comonomers, d: 4.09 ppm (s, 2H, OCH_2) and 0.95 ppm (t, 3H, CH_3) for BA and 4.47 ppm (t, 1H, CH) for VC (referred to solvent).

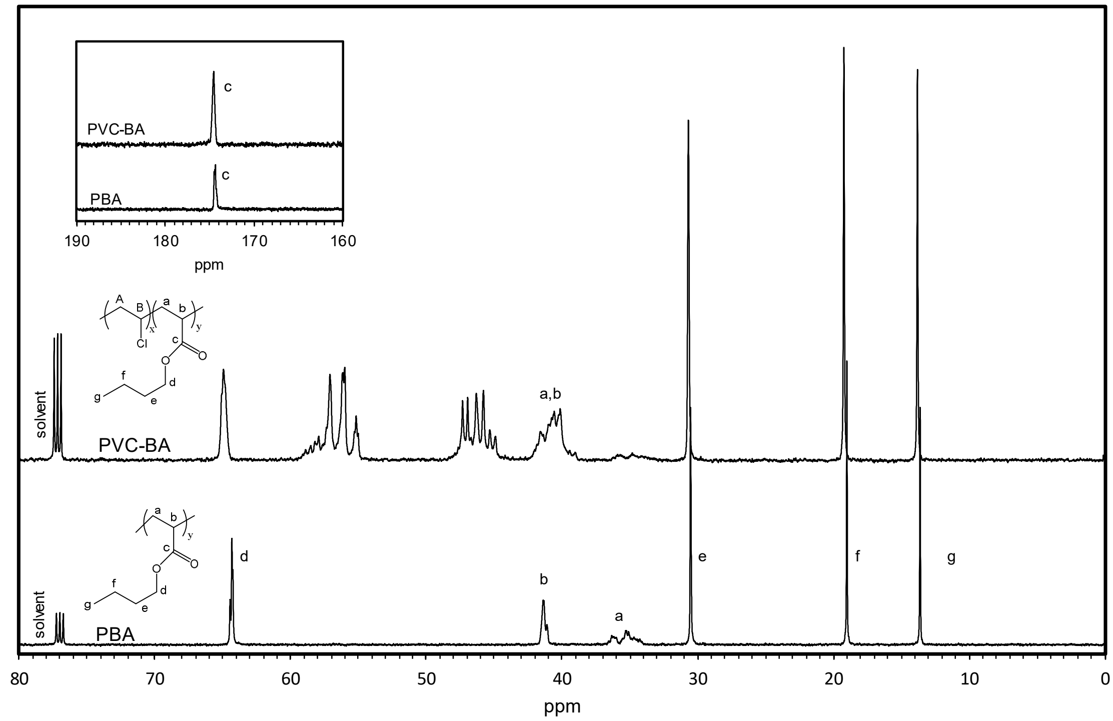

Figure S3. Comparison between ^{13}C-NMR spectra for synthesized PVC-BA copolymers and PBA homopolymer. The shifting of the lines from 35 ppm to 40 ppm indicates the presence of alternative AVA and/or VAV sequences as predominant structures instead of AAA homopolymer sequences. However, although the intensity is very low, the presence of such homopolymer sequences can not be discarded.
